# From pathogenesis to prevention: an update on the management of obesity and its associated comorbidities in cats

**DOI:** 10.3389/fvets.2026.1797197

**Published:** 2026-04-07

**Authors:** Huakai Wang, Weiwei Wang, Yuqiang Zhang, Jiahang Yao, Yiran Liu, Li Pan, Ran Wang, Jianmei Wang, Zhaofei Xia, Lishui Chen, Wei Xiong

**Affiliations:** 1 Food Laboratory of Zhongyuan, Luohe, China; 2 College of Food and Bioengineering, Zhengzhou University of Light Industry, Zhengzhou, China; 3 Key Laboratory of Precision Nutrition and Food Quality, Department of Nutrition and Health, China Agricultural University, Beijing, China; 4 College of Veterinary Medicine, China Agricultural University, Beijing, China

**Keywords:** associated comorbidities, feline obesity, pathophysiology, prevention, risk factor, weight loss

## Abstract

Overweight and obesity represent the most common nutritional disorder in domestic cats and constitute a significant global health issue. In this review, we synthesize current knowledge on the determinants, diagnosis, pathophysiology, complications, and comprehensive management of feline obesity. Feline overweight and obesity have a complex and multifactorial pathogenesis, arising from an interplay of intrinsic factors (e.g., genetic predisposition, age, sex, neutering status) and extrinsic factors (e.g., environment, owner behavior). The condition is characterized by a state of chronic low-grade systemic inflammation, endocrine dysregulation, insulin resistance, and hyperlipidemia, which collectively elevate the risk of numerous comorbidities, including diabetes mellitus, osteoarthritis, urinary tract disorders, dermatopathies, cardiomyopathy, and respiratory diseases, ultimately compromising life expectancy. This means that early detection and examination of excess body weight are crucial to treatment and prevention; at the same time, weight loss should be centered around personalized nutritional intervention, combined with behavioral correction measures such as regular feeding schedules and increased physical exercise. Furthermore, maintaining good communication between clinicians and the pet owners, as well as continuous monitoring, is the key to achieving effective weight loss. Future research is needed to move beyond current reactive models and embrace a focus on metabolic health over weight, prediction over reaction, and pathogenesis over symptomatology, aiming for preemptive strategies that improve feline healthspan.

## 1 Introduction

Overweight and obesity is a chronic condition that has a complex pathogenesis that is mainly manifested by excessive deposition of adipose tissue. Feline overweight prevalence has been reported to range between 11.5–63% to make it a critical health issue on the global scale (1, 2). Numerous comorbidities accompany this disease, including diabetes, orthopedic disorders, urinary tract diseases, skin diseases, respiratory diseases, and cardiomyopathy (3, 4). While the multifactorial etiology—encompassing genetic predisposition, neutering status, and obesogenic indoor environments—has been previously described (5, 6), a critical gap remains in translating this knowledge into effective, long-term management. A major barrier is the persistent disconnect between veterinary assessment and owner perception, with many caregivers failing to recognize overweight conditions in their own pets. This review aims to bridge this gap by providing a comprehensive, updated synthesis of the evidence base for feline obesity management. Unlike previous reviews that may focus on isolated aspects, we uniquely integrate recent advances across the entire spectrum of the condition: from a detailed examination of pathophysiological mechanisms (including adipose tissue dysfunction and metabolic inflammation) to evidence-based diagnostic and therapeutic strategies. Furthermore, we critically evaluate emerging paradigms, such as the potential for pharmacotherapy and the shift from reactive treatment to predictive, preventive metabolic health management. By consolidating this knowledge, our objective is to equip veterinary practitioners and researchers with a practical tool to not only manage but also proactively prevent obesity, thereby enhancing the quality of life and longevity of affected cats.

## 2 Risk factors

**Figure 1:**
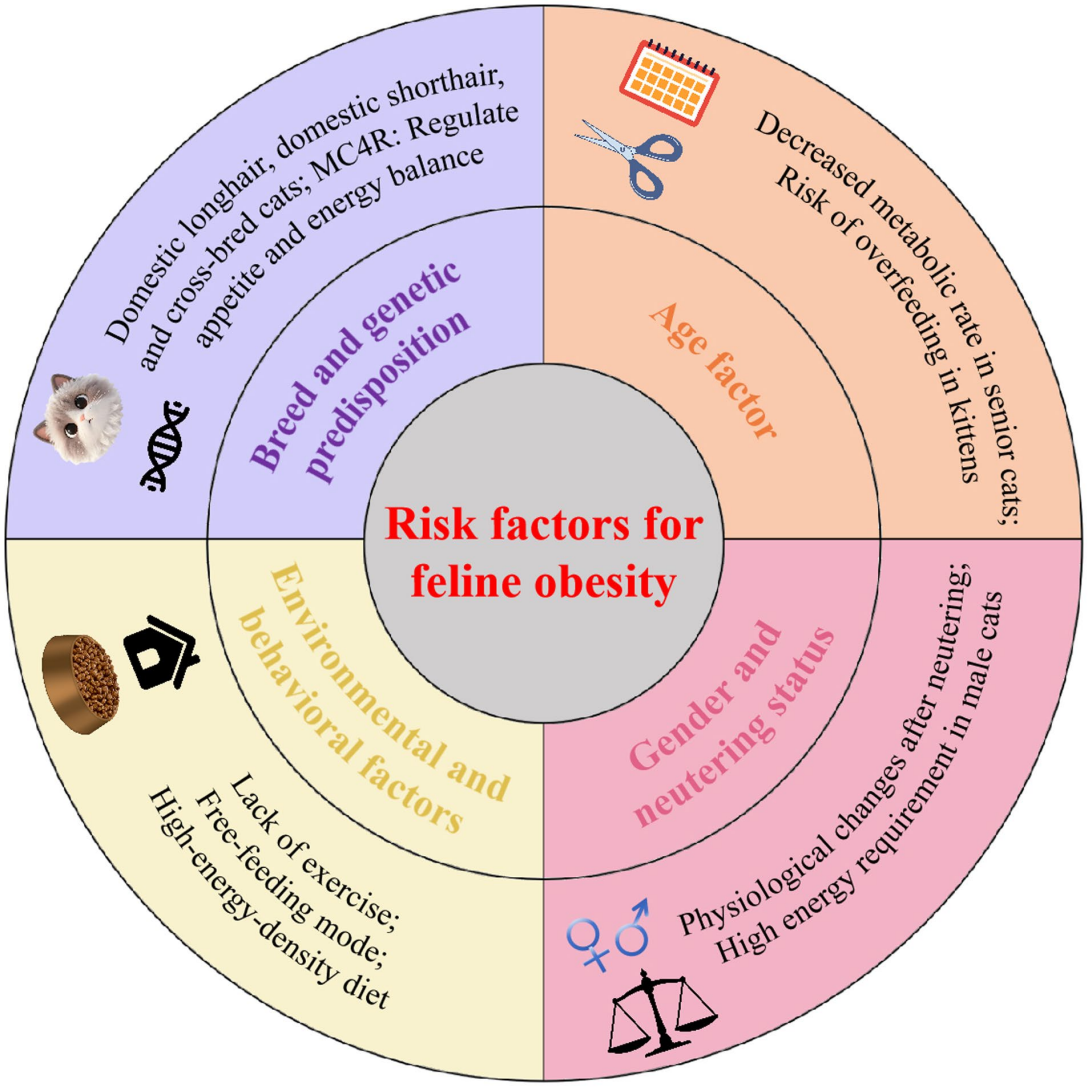
Multidimensional risk factors for obesity in cats. This figure illustrates the key risk factors associated with feline obesity: breed and genetic predisposition, age, sex, and neutering status, and environmental and behavioral factors.

There are several risk factors associated with feline obesity, including genetic vulnerability, age, sex, neutering, and numerous environmental and behavioral interdependence factors (Figure 1).

### 2.1 Breed and genetic predisposition to overweight

The basic etiology of obesity is energy imbalance; however, the genetic makeup of an animal is a significant determinant of the predisposition to an increase in weight in a consistent environmental stimulus. This inherent risk can be observed in breed-specific tendencies, although the evidence regarding specific breeds as risk factors remains conflicting. Some studies have reported a higher prevalence of obesity in domestic longhair, domestic shorthair, and cross-bred cats relative to pure-bred cats, such as Abyssian, Cornish Rex, and Sphynx cats (7, 8). In contrast, other studies reported that there is no significant breed-specific correlation (9, 10). Corbee provided a possible explanation, which was inter-breed discrepancies caused by the aesthetic standard (11). Thus, addressing this genetic predisposition may require reconsidering the aesthetic standards among breeders and judges combined, whereby functionality and health in general should be given preference over body mass.

While breed can be a proxy for genetic risk, a deeper understanding requires examining specific molecular mechanisms. Insights from human genome-wide association studies have validated the relationship between several genes, including MC4R, BDNF, LEP, LEPR, and FTO, that are highly related to the risk of obesity in humans (12, 13). In mammals, the G-protein-linked receptor MC4R plays a central role in the hormones connected with the regulation of appetite and energy (14). Current feline studies have found single-nucleotide polymorphisms (SNPs) in the MC4R gene that have important links with body condition that support its role in predisposing individuals to obesity (6). Apart from direct genetic mutations, epigenetic mechanisms such as DNA methylation also participate in the interaction between environmental factors and gene expression, which can change the obesity susceptibility by altering gene regulation without changing the underlying DNA sequence (15).

### 2.2 Age

The risk of feline obesity exhibits a distinct age-related pattern, with evidence suggesting its onset can occur very early in life. Studies indicate that overweight and obesity can be present in cats as young as 12–13 months, particularly associated with indoor confinement and consumption of dry diets (16). A recent large-scale study involving over 1.3 million cats from primary care practices in the USA provided detailed prevalence data across the entire lifespan. This study revealed that the prevalence of overweight/obese body condition increases progressively from late growth (10.7% overweight, 0.4% obese) through young adulthood (36.2% overweight, 3.6% obese) and adulthood (47.2% overweight, 13.9% obese), peaking during the mature life stage (44.8% overweight, 21.7% obese), before declining in senior cats (32.0% overweight, 12.6% obese) (17). This peak period is likely attributable to an age-related decline in metabolic rate, coupled with reduced activity levels, potential changes in nutrient digestibility, and energy intake that often fails to adjust accordingly (18, 19). Furthermore, Mizorogi et al. confirmed positive correlations between advancing age and increases in body weight, body condition score (BCS), serum glucose, amyloid A, triglycerides, and blood urea nitrogen in cats (20). Importantly, the study by Montoya et al. also demonstrated that cats identified as overweight or obese during growth had 1.52 times higher odds of remaining overweight or obese in adulthood, highlighting the critical importance of early-life weight management (17).

### 2.3 Sex

Some studies suggested that male cats may have a higher risk of being overweight compared to female cats, which is often attributed to their higher energy maintenance requirements and generally larger body size (10, 21). However, this association varies across different populations and studies. This sex difference is usually explained as the fact that male cats have a higher baseline energy requirement, and if the feeding management is improper, it is more likely to result in a positive energy balance (22, 23). Furthermore, this risk may be compounded by owner misperception, as caregivers often struggle to accurately assess their cat’s body condition and may inadvertently overfeed, particularly in larger or male cats where the visual discrepancy between ideal and actual weight is less obvious to the owner.

### 2.4 Neutering status

One of the most evident and well-supported risk factors for feline obesity is neutering. The underlying mechanisms involve profound hormonal alterations that disrupt the central regulation of energy homeostasis, primarily driven by the removal of gonadal steroids (testosterone in males, estradiol in females). These hormones are essential for modulating appetite and energy expenditure. Their absence leads to a reduced basal metabolic rate (BMR) and increased appetite. Specifically, the loss of estrogen’s anorexigenic (appetite-suppressing) effect and testosterone’s role in maintaining lean body mass and metabolic rate are key contributors. Consequently, this hormonal vacuum dysregulates hypothalamic pathways involving neuropeptides such as leptin and ghrelin, resulting in a persistent state of positive energy balance if caloric intake is not adjusted (24, 25). While both sexes are at increased risk, some studies indicate that neutered male cats may experience a more pronounced increase in food intake, whereas neutered females might exhibit a relatively greater decline in BMR (26–28). These differences are hypothesized to stem from the distinct baseline roles of testosterone versus estrogen, though findings across studies are not entirely consistent (24, 25). Neutering is also associated with behavioral changes, including reduced roaming and general activity levels, which further contribute to a decrease in total daily energy expenditure (29). The convergence of increased appetite, lowered metabolic rate, and reduced activity creates a perfect storm for weight gain. This risk is particularly acute during the first year post-procedure, making this period crucial for proactive weight monitoring and dietary management (25). It is important to emphasize that neutering itself is not a direct cause of obesity but rather a potent modifier of physiological set points. Consequently, it necessitates direct and sustained owner intervention through lifelong adjustments in dietary care and feeding habits to prevent excessive weight gain and its associated health consequences.

### 2.5 Environmental and behavioral factors

Environmental and behavioral factors that are linked to feline obesity include eating habits, feeding schedules, indoor house confinement, and sedentary nature (21, 30, 31). Critically, the cat-owner relationship—encompassing owner perception, feeding behavior, and compliance—is a fundamental determinant that influences all these factors. In a study conducted by Öhlund et al. (18), the authors found that the type of diet was correlated with feline obesity, and cats fed a predominantly dry diet had a 2.4 times higher risk compared to cats fed a predominantly wet diet. A study by Rowe et al. demonstrated that cats fed a diet consisting of more than 50% dry food from an early age (i.e., post-weaning) had a 79% higher risk of being overweight or obese by around 1 year of age. This association is possibly linked to the lower moisture content of dry diets, which may influence satiety, gut fill, and energy density (16). Another well-established risk factor is ad libitum feeding, particularly of highly palatable and energy-dense foods, which can disrupt natural satiety signals and lead to chronic over-consumption (32, 33). Furthermore, exclusive indoor housing, particularly in apartments or homes without access to outdoor environments, restricts opportunities for energy expenditure through natural behaviors such as exploration, roaming, and interspecific interactions (34). Consequently, owner-related factors are central to both the development and management of obesity. This results in the necessity to increase the education of owners, specifically addressing misconceptions about ideal body condition, the emotional drivers of overfeeding (e.g., using food as affection), and the importance of adherence to veterinary plans. This education should highlight the significance of regular weighing and tailoring energy intake to each cat’s individual demands, taking into consideration neutering status, age, and activity level (33).

## 3 Diagnosis and assessment

The accuracy of body composition assessments is critical in making an optimal body weight and energy assessment, hence playing a critical role in the diagnosis and assessment of excess body weight. A clear distinction should be made between overweight, defined as body weight exceeding the ideal range, and obesity, a pathological state characterized by excessive adipose tissue accumulation that impairs health. Body composition is typically conceptualized as comprising two main components: fat mass and fat-free mass (i.e., muscle, organs, and water) (35). This two-compartment model provides a useful physiological framework for understanding energy balance and metabolic health. Its primary advantage lies in its simplicity and direct relevance to energy stores and metabolic function. However, a limitation is that it does not provide information on the distribution of fat (e.g., visceral vs. subcutaneous) or the quality of lean tissue, which can be important for assessing metabolic risk. A variety of methods are available to estimate these components, each with specific strengths and limitations in clinical and research settings.

### 3.1 Body weight

Body weight is an applicable and measurable parameter that can be used to track the pattern of weight change and to evaluate weight-loss programs (36, 37). However, excessive use of body weight is inconvenient, as it does not indicate fat percentage and differences between muscle or adipose tissue (38). It also ignores inherent variations stemming from breed, sex, and skeletal frame size (39). Nevertheless, when monitoring an individual cat over time, serial body weight measurements provide an excellent and practical tool for tracking body fat changes during adulthood, assuming no concurrent conditions that might independently alter muscle mass (e.g., chronic disease, sarcopenia). Therefore, body weight is most informative when tracked over time and interpreted alongside other assessments like BCS.

### 3.2 Body condition score

**Figure 2:**
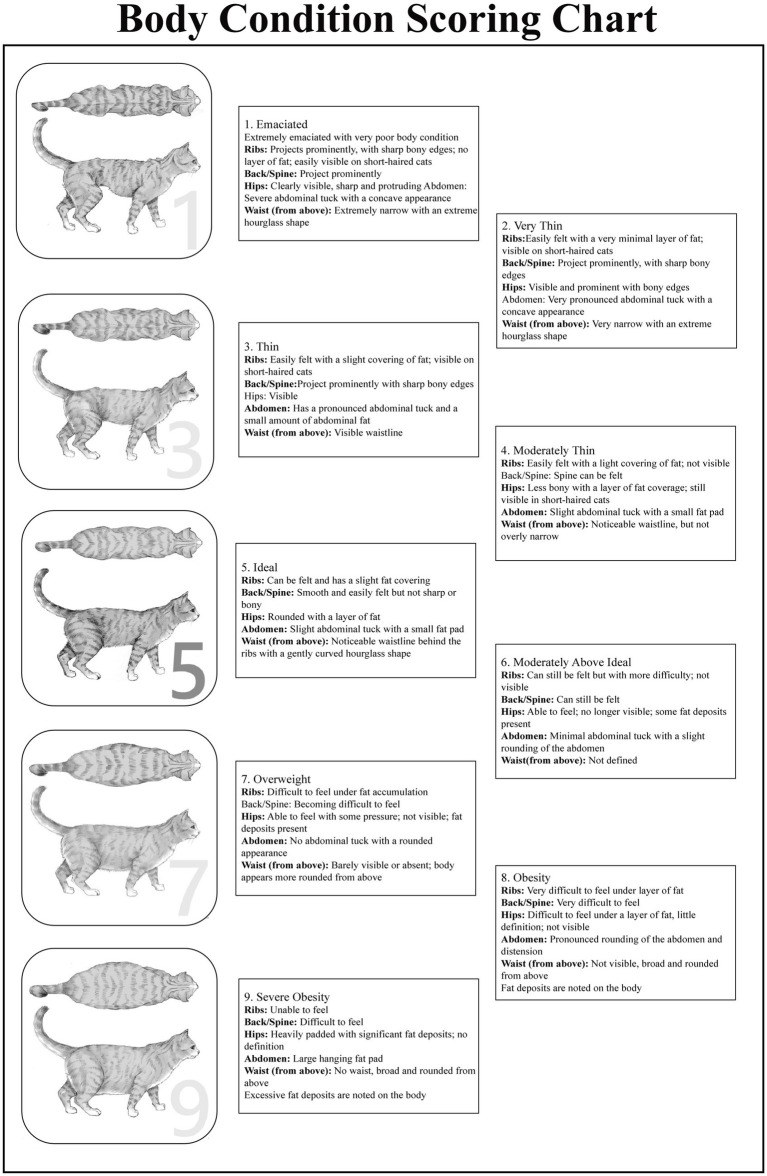
Feline body condition scoring scale (9-point system) (41).

Body condition score is a semi-quantitative measure of the state of nutritional status through visual examination and palpation experience, widely used in veterinary practice. The three primary indicators are the amount of fat covering the ribs, the presence and definition of a waist, and the size of the abdominal fat pad (38). BCS systems are based on a 5-point scale or a 9-point scale; the 9-point scale has been verified to be able to estimate the body fat percentage of a cat with good reproducibility and has shown a high correlation with data offered by dual-energy X-ray absorptiometry (DXA) (35, 40, 41). The 9-point system, which has been validated, used a 1 to indicate emaciation, 5 represents a perfect body, 6–7 indicate overweight, and 8–9 indicate obesity (38, 41), and The standard is shown in Figure 2. However, it is noteworthy that the interpretation of a BCS of 6/9 may be context-dependent. For instance, Teng et al. (42) reported increased survival in middle-aged cats with a BCS of 6/9 compared to those with a score of 5/9, suggesting this score may be acceptable or even beneficial for some populations. To support decision-making, a series of anatomical outline diagrams is provided to illustrate the visual characteristics of typical cats at each score (Figure 2) (41). It is also important to acknowledge factors that can affect BCS consistency in practice. Variability can arise from differences in experience among veterinary professionals, and owners’ visual perception of their cat’s body condition often differs from clinical assessment, which may impact early diagnosis and monitoring adherence. Inter-observer assessments have shown good correlation in BCS results (41). However, BCS mainly assesses the degree of subcutaneous fat coverage and does not directly quantify muscle mass. This limitation means that in individuals who are overweight or obese, the occurrence of muscle-depleted obesity (i.e., increased fat accompanied by muscle loss) may be overlooked, thereby leading to inaccurate estimations of body fat percentage (36, 43). Domestic cats owned by clients are likely to have higher percentages of fats than colony cats with the same BCS due to a sedentarized lifestyle and loss of mass (38). However, cats with a BCS of 9 can have very differing levels of real body fat, which indicates the inappropriateness of the scale in deeply obese cats. Regardless of these drawbacks, BCS could be of importance in the assessment and monitoring of single cats because of its simplicity, intuition, and reproducibility in clinical practices.

### 3.3 Feline body mass index

Feline body mass index (BMI) is a quantitative parameter that classifies the cats into underweight, normal, overweight, and obese depending on the weight and height. Several formulas for calculating feline BMI have been proposed in the literature; the following represents one commonly cited example (44). All measurements should be taken while the cat is in a standing position, with its limbs perpendicular to the ground and its head held upright. The percentage of body fat is determined by the following Equation 1:


$$\%\text{Body}\;\mathrm{fat}=(\frac{(\frac{\text{Ribcage}}{0.7067})-\mathrm{LIM}}{0.9156})-\mathrm{LIM}$$ (1)

Note: Ribcage-Circumference at the 9th thoracic vertebra (or the 5th lumbar vertebra) (unit: cm); Leg index measurement (LIM)-The distance between the patella (knee) of the left hind limb and the calcaneal tuberosity (tarsal joint) is measured in cm.

Hoelmkjaer and Bjornvad (7) reported that this method yields significantly greater accuracy than BCS for predicting body fat percentage and shows a strong correlation with body weight. Nevertheless, the practical utility of feline BMI in clinical application remains limited, as accurate measurement requires the cat to maintain a specific standing position with limbs perpendicular to the ground and head upright—a posture that many cats, particularly those that are anxious or uncooperative, may not reliably sustain during a veterinary consultation.

### 3.4 Dual-energy X-ray absorptiometry

Apart from bone density measurement, DXA is also able to examine the composition of the body: percentage fat, lean soft tissue, and bone (45). It works by measuring the differential attenuation of two low-energy X-ray beams as they pass through tissues of different density. Speakman et al. showed concordance of DXA with chemical analysis in a validation study using both cats and dogs weighing 1.8 and 22.1 kg (46). Nevertheless, such a pattern was not observed in beef, lard and water mixtures, suggesting that the instrument had a tendency to overestimate fat in fine body as the percentage of water content went up, meaning that water percentage plays a significant role in DXA accuracy (46). Additionally, this method has limitations such as inaccuracy with extreme body types, high equipment costs, and the need for specialized expertise, which currently restricts its application primarily to the field of scientific research.

### 3.5 Bioelectrical impedance

Bioelectrical impedance (BIA) determines the proportion of fats and lean tissues in the body by using the difference between the electrical conductivity of adipose tissue and lean tissue, using a low-frequency current through electrodes and the predictive model (47). An increase in muscle mass, which is a predictor of higher water content, decreases electrical resistance, but increased adiposity increases resistance (48). Stanton et al. had shown a reliable prediction of total body water, potassium, and fat-free mass with a high level of correlation to chemical analysis (49). The accuracy is determined by the hydration, location of the electrode, the amount of hair on the body, the size of the body, breed, and whether the animal is full or hungry. Nevertheless, the fact that specific predictive equations are used and high technical sophistication is a requirement is also detrimental to mass application.

### 3.6 Ultrasonography

Body composition measurement by ultrasonography varies based on the differences in the speed of sound propagation and acoustic impedance between high-frequency sound across different tissues using ultrasonography to assess subcutaneous adiposity, muscle thickness, and quality (50). The method is fast and non-radiation as opposed to DXA (51), and it has been used in working dogs to monitor muscular changes and training outcomes. Nevertheless, the ultrasonography does not provide whole-body composition and is not validated in feline evaluation.

### 3.7 Other methods

Both computed tomography (CT) and magnetic resonance imaging (MRI) are gold standard techniques in the field of medical imaging, providing high-resolution cross-sectional images of the body, thereby enabling precise quantification of body composition such as fat, muscle, and bone (52, 53). However, for the detection of obesity in cats, the high costs, necessity of anesthesia, and limited clinical accessibility make these methods far less practical and efficient than veterinary physical examination, specifically BCS. Consequently, they are primarily confined to scientific research rather than routine clinical diagnosis.

## 4 Pathophysiology

**Figure 3:**
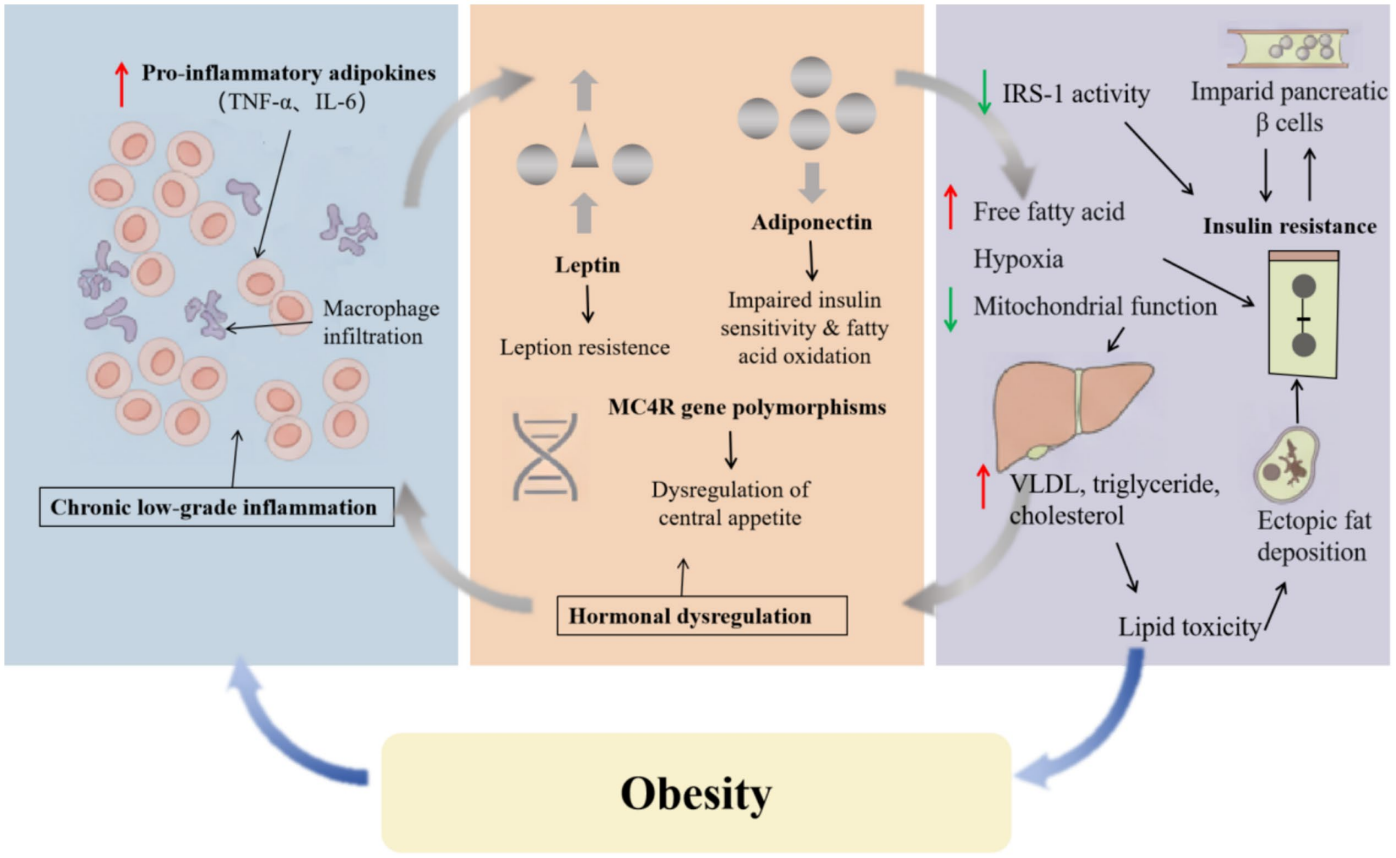
The pathophysiological mechanism of feline obesity. This figure illustrates the vicious cycle mechanism of obesity and hormonal imbalance.

The pathophysiology of feline obesity extends far beyond simple energy imbalance; it is a multifaceted metabolic disorder characterized by adipose tissue dysfunction, hormonal dysregulation, and global metabolic changes. All these interrelated processes form a self-perpetuated vicious cycle that not only sustains obesity but also leads to its associated comorbidities, indicating the need to address the underlying metabolic dysfunctions that drive and maintain obesity, rather than focusing solely on weight reduction as the primary therapeutic endpoint (Figure 3).

### 4.1 Adipose tissue dysfunction and metabolic inflammation

In feline obesity, the fat tissue not only exceeds its traditional role as an energy storage organ, but also undergoes significant pathological remodeling. Critically, the metabolic impact differs by location: visceral (abdominal) fat is more strongly associated with dysfunction than subcutaneous fat. Although much of our detailed understanding of adipose tissue pathophysiology derives from human and rodent studies, similar processes are believed to occur in cats. A state between hypertrophy and hyperplasia controls the structure and homeostasis of adipose tissue, and its balance is disrupted by body weight variations (54). With progressive adiposity, adipocytes become hypertrophic and hypoxic, which results in the expression of pro-inflammatory adipokines, including TNF-*α* and IL-6, while reducing the secretion of anti-inflammatory factor adiponectin, causing a chronic low-grade inflammatory state (55). Macrophage infiltration, a well-characterized feature of adipose tissue inflammation in other species, likely amplifies the inflammatory response in obese cats, although direct feline studies on this specific mechanism remain limited (56). These processes help explain the clinical spectrum of obesity. The distinction between metabolically healthy obesity (MHO) and metabolically unhealthy obesity (MUO) may relate to differences in fat distribution and adipokine profiles. Supporting the central role of lipid dysregulation, Okada et al. (57) highlighted that in cats, elevated triglycerides are a more reliable marker of metabolic risk than cholesterol. These interrelated mechanisms thereby establish a crucial pathological link between obesity and its complications.

### 4.2 Hormonal dysregulation

The complexity of hormonal imbalance is one of the characteristics of feline obesity, and it can be classified as interference with the normal processes that regulate appetite, metabolism, and energy balance. In obese cats, increased circulating leptin concentrations have been documented, and it is hypothesized that, similar to other species, they develop leptin resistance—a state where elevated leptin fails to exert its normal satiety-signaling effects in the hypothalamus, potentially promoting excessive eating and reduced energy expenditure (58). At the same time, obesity can inhibit the production of adiponectin, a hormone that enhances insulin sensitivity and fatty acid oxidation, which severely disrupts the balance of glucose and lipid metabolism (59). Furthermore, these hormonal imbalances will further lead to endocrine system disorders, resulting in weight gain and metabolic dysfunction. This imbalance is also regulated by genes, as there are genetic variations in the MC4R gene polymorphisms associated with an increase in body mass index, and these variations are likely to cause an imbalance in central appetite (60).

### 4.3 Insulin resistance and dyslipidemia

Inflammation and endocrine disorders can lead to metabolic defects, primarily insulin resistance and dyslipidemia. Notably, cats appear to be particularly prone to developing insulin resistance compared to other species like dogs. This heightened susceptibility may be attributed to several inherent physiological factors. For instance, healthy cats have been shown to possess lower circulating concentrations of adiponectin, an insulin-sensitizing adipokine, than dogs (61). Furthermore, comparative studies suggest that cats have lower expression levels of key genes involved in the insulin signaling pathway and lipid metabolism compared to their canine counterparts, which may predispose them to metabolic dysregulation under conditions of positive energy balance (62–64). The molecular mechanisms underlying insulin resistance have been extensively studied in humans and rodent models. On one hand, pro-inflammatory cytokines utilize signaling pathways to inhibit the activity of insulin receptor substrate-1 (IRS-1), thereby blocking insulin signaling—a mechanism well-characterized in rodent and human studies (65); On the other hand, elevated levels of free fatty acids, hypoxia, and impaired mitochondrial function further lead to impaired glucose uptake in muscles and uncontrolled glucose production in the liver, as demonstrated in both human and animal models (66–69). While direct evidence for these specific pathways in cats is limited, it is likely that similar mechanisms contribute to the insulin resistance observed in obese felines. In the enlarged adipose tissue, fat breakdown accelerates, and a large amount of free fatty acids enters the portal venous circulation. This will increase the synthesis and secretion of very low-density lipoprotein (VLDL) in the liver, thereby leading to an increase in triglyceride and cholesterol levels in the circulation (70). This excessive lipid content can cause lipid toxicity, leading to ectopic fat deposition in the liver (which is prone to lipid accumulation) and skeletal muscles, which will further exachaoerbate insulin resistance and damage the function of pancreatic *β* cells (71, 72).

## 5 Associated comorbidities

**Figure 4:**
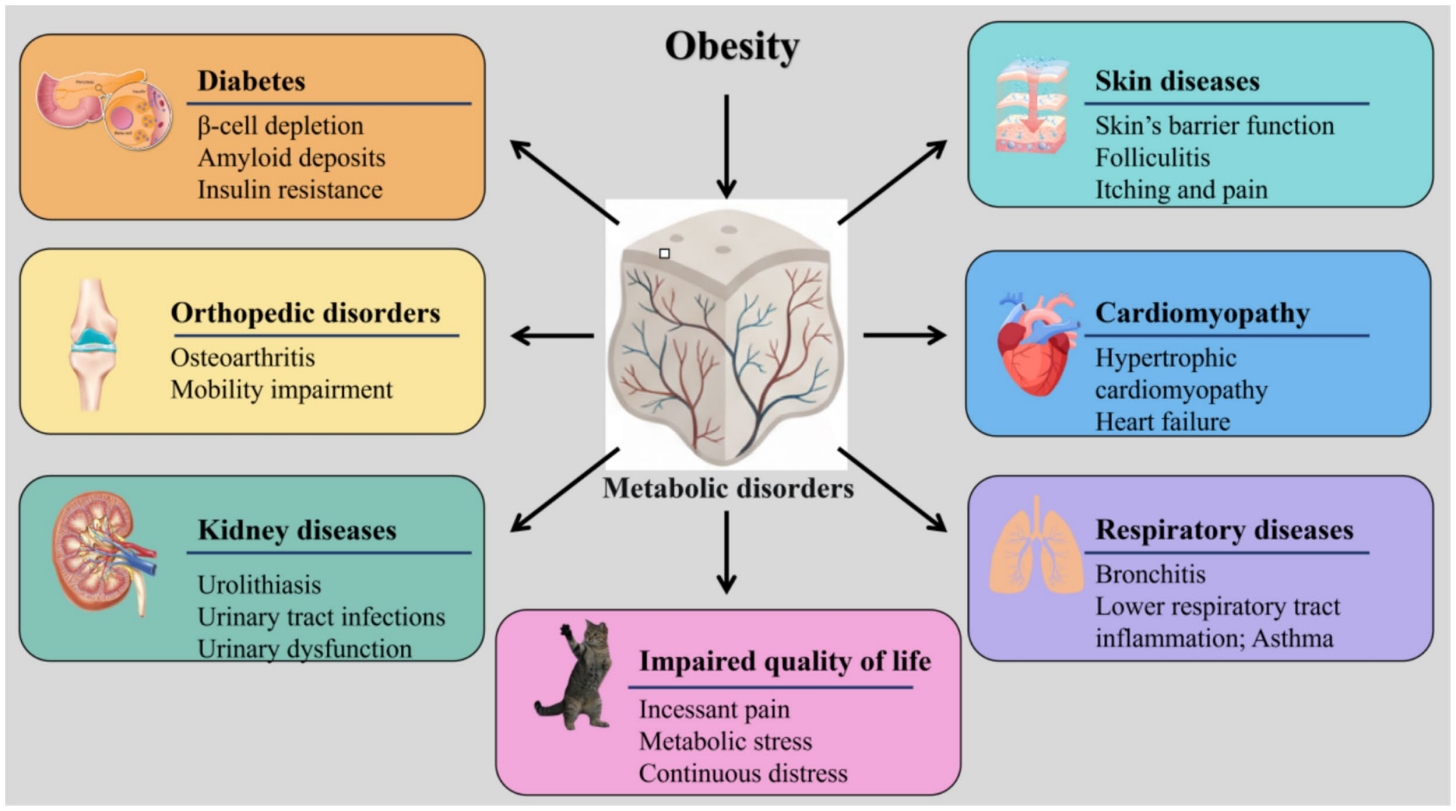
Feline obesity-related complications network. This figure illustrates the key complications associated with feline obesity: diabetes, orthopedic disorders, kidney diseases, skin diseases, cardiomyopathy, respiratory diseases, and impaired quality of life.

As a metabolically active endocrine organ, excess adipose tissue in obesity results in various complications through disrupting normal physiological homeostasis by secreting bioactive substances, such as adipokines (Figure 4).

### 5.1 Diabetes

Obesity is the major risk factor for the development of diabetes mellitus in cats, mirroring the pathophysiology of human type 2 diabetes (73). The relationship is bidirectional and self-perpetuating: obesity-induced metabolic dysfunction leads to insulin resistance and *β*-cell impairment, which in turn exacerbates weight management challenges. The central mechanism linking adiposity to diabetes is adipose tissue dysfunction, characterized not merely by excess fat, but by pathologic remodeling.

The expanded, dysfunctional adipose tissue becomes a source of chronic, low-grade inflammation and altered adipokine secretion (e.g., increased leptin, decreased adiponectin) (74, 75). Based on extensive research in human and rodent models of obesity, these factors are known to activate intracellular stress pathways (e.g., NF-κB, JNK) in insulin-sensitive tissues, directly impairing insulin signaling; it is plausible that similar pathways are operative in cats, though direct feline studies are lacking (76). Crucially, the distribution of adipose tissue is more determinative than total fat mass. In abdominal/visceral obesity, lipolysis releases excessive free fatty acids (FFAs) and leads to ectopic fat accumulation in liver and muscle (77). In the liver, this causes steatosis and lipotoxicity, further impairing insulin’s suppression of gluconeogenesis (78, 79). In muscle, lipid intermediates like diacylglycerol (DAG) activate PKCε, disrupting insulin-stimulated glucose uptake—a pathway primarily elucidated in human and rodent studies (76). These mechanisms, while well-established in other species, provide a framework for understanding the pathogenesis of feline diabetes, although species-specific confirmation is needed. The resultant hyperglycemia and elevated FFAs create a glucolipotoxic environment for pancreatic *β*-cells, leading to oxidative stress, endoplasmic reticulum stress, amyloid deposition, and ultimately, apoptosis and functional decline (79). This creates a vicious cycle: insulin resistance demands more insulin, straining the β-cells, whose failure then deepens hyperglycemia.

### 5.2 Orthopedic disorders

Obese cats are almost twice as likely to exhibit symptoms associated with joint disease compared to cats of normal weight, which was initially attributed to abnormal mechanical loading on the joints (80). However, studies have found that joint disease also occurs in non-weight-bearing joints in obese individuals, suggesting that obesity-related biochemical factors may also contribute to the development of joint pathology (81). Toda et al. also demonstrated that reducing body fat is more critical for alleviating symptoms of knee osteoarthritis than simply reducing body weight or other obesity-related metrics (82). In human medicine, inflammatory mediators (such as TNF-*α* and IL-6) and adipokines (including leptin and adiponectin) released during obesity have been implicated as potential pathophysiological mechanisms contributing to osteoarthritis (83). Although direct evidence for these biochemical pathways in feline osteoarthritis is limited, given the conserved nature of inflammatory responses across mammals, these mechanisms are likely applicable to the feline species as well. Further research is needed to confirm the specific role of adipokine-mediated inflammation in feline joint disease.

### 5.3 Urinary tract disorders and kidney diseases

The obesity of cats is closely related to the health risks associated with the urinary system, particularly an increased risk of urolithiasis and lower urinary tract diseases (18). Previous research found that sterilized cats have an 8.3 times higher risk of developing urinary tract stones compared to non-sterilized cats, and overweight cats have a risk of lower urinary tract diseases that is 4 times higher than that of cats of normal weight (84). The core mechanism involves obese cats not only consuming more stone-prone minerals but also having reduced physical activity, which decreases their water intake and thereby concentrates their urine. This problem is further exacerbated by neutering and an indoor lifestyle, which collectively reduce urination frequency, significantly promoting stone formation and urinary tract infections (85, 86). Furthermore, the urethra and penis can also be squeezed by the surrounding fat, causing more damage to urinary dysfunction. Contrary to humans, a direct relationship between obesity and chronic kidney diseases has not been defined in felines; a healthy-cat study did not show any relationship, and mild hyperglycaemia could disrupt kidney physiology-markers (87, 88).

### 5.4 Skin diseases

Feline obesity induces chronic inflammation and endocrine disorders, impairing the skin’s barrier function and increasing susceptibility to pathogens and allergens (89). While similar associations between obesity and skin disease are well-documented in humans (90), the direct evidence in cats supports this relationship (89). Moreover, large body mass prevents good grooming, increases the risk of infection, including folliculitis, and the abundant skin folds increase the frequency of bacterial dermatitis. Overweight or obese cats exhibit a 2.3-fold higher risk of nonspecific, non-allergic skin conditions compared to healthy-weight cats (89, 91). Itching and pain decrease activity and increase stress in a vicious cycle, which further promotes weight gain. Additionally, corticosteroid-based therapies are potentially stimulating to appetite, which leads to additional weight gain (92). Therefore, the abnormal appetite and weight gain can be a side effect of the treatment of skin diseases, as well as a consequence directly associated with the skin diseases themselves.

### 5.5 Cardiomyopathy

Cardiomyopathy, particularly hypertrophic cardiomyopathy (HCM), is the most common cause of heart disease in cats, accounting for over 60% of all feline cardiac cases (93). Unlike in humans, obesity carries a low risk of triggering atherosclerotic coronary artery disease in felines. While this has been attributed to a favorable high-density lipoprotein (HDL) to low-density lipoprotein (LDL) ratio in carnivores, this explanation is primarily based on studies in dogs, and direct confirmation of this mechanism in cats is lacking (94). Although obese cats exhibit elevated VLDL levels similar to humans, they do not develop hypertension or atherosclerosis, suggesting the presence of other protective mechanisms against atherosclerosis in cats (93, 95). In studies focused on feline HCM, body weight was not identified as a risk factor, and there was no significant difference in BCS between HCM-affected cats and control cats, despite HCM cats often having larger frames (96, 97). It is worth noting that although obesity itself is not regarded as a direct causative factor for HCM, Finn et al. found in a study of cats with heart failure that the survival rate of overweight cats was lower than that of cats with a moderate weight, indicating that obesity may have an adverse effect on the prognosis of heart failure (98).

### 5.6 Respiratory diseases

The relationship between feline bronchitis, an inflammatory lower respiratory condition and obesity is uncertain. Garcia-Guasch et al. found that there were no differences in tidal volume, inspiratory time, partial pressure of carbon dioxide (PCO₂), and respiratory rate between healthy-weight and obese cats (99). Similarly, the results of Champion et al. also showed that no differences between overweight and normal-weight cats across multiple respiratory indicators, including tidal volume, inspiratory time, peak pressure, respiratory rate, end-tidal carbon dioxide partial pressure (PETCO₂), and arterial partial pressure of oxygen (PaO₂) (100). On the contrary, Garcia-Guasch et al. showed lower tidal volume, minute ventilation per kilogram of body weight, peak inspiratory and expiratory flow rates in obese cats (101). Caro-Vadillo et al. also found a reduced tidal volume, minute ventilation and maximal inspiratory and expiratory flow in overweight cats with no alteration in the severity of bronchoconstriction, indicating that pulmonary impairment is not caused by exacerbation, but it can be caused by the changes in metabolism (102). It is worth noting that, while lower respiratory tract inflammation and asthma are relatively common in cats, upper respiratory tract infections have not been identified as a relevant risk factor (93). In addition, some studies have suggested that obesity may exacerbate bronchial inflammation mediated by neutrophils by regulating adiponectin and leptin levels, which is a potential mechanism by which obesity affects the respiratory health of cats (102).

### 5.7 Impaired quality of life

This general comorbidity is a summative load of multifactorial conditions- osteoarthritis, grooming problems, insulin resistance, and systemic inflammation that all contribute to degrading physical and mental health in a cat. Incessant pain, metabolic stress, and continuous distress considerably affect the quality of life of the cat and restrict its ability to engage in natural behaviors, which include jumping, playing, and grooming. Consequently, obese cats not only have a reduced lifespan but also a significantly diminished daily experience. Although validated quality-of-life studies specifically in obese cats are currently lacking, this observation aligns with findings in dogs, where such assessments have demonstrated significant impairment in obese individuals that improves following weight loss (103). It is reasonable to hypothesize that a similar impact on quality of life occurs in cats, given the shared physiological and behavioral consequences of obesity across species. This prolonged state of poor health is often overlooked by the owners until the cat successfully loses weight, at which point it becomes more energetic and active. Therefore, management of obesity is one of the most effective interventions to increase the feline lifespan and welfare.

## 6 Management strategies

**Figure 5:**
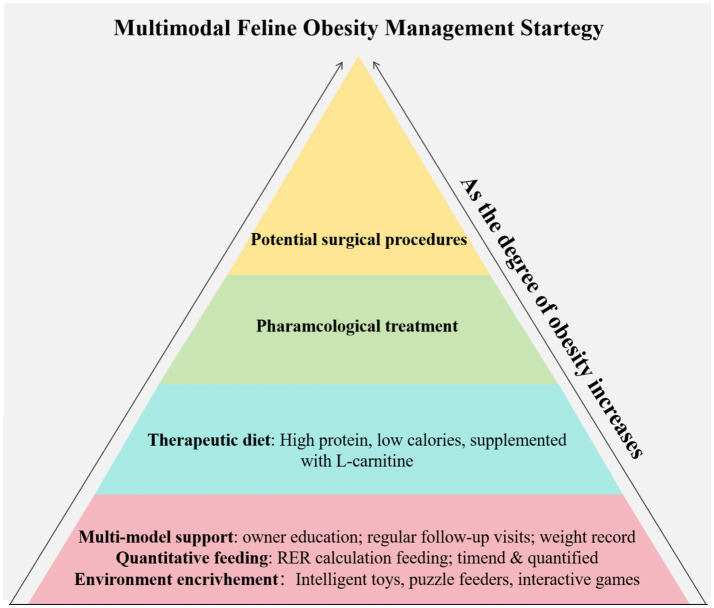
Multimodal management strategy for obesity in cats.

The treatment of human obesity follows a multimodal management approach, encompassing lifestyle interventions (diet and exercise), behavioral modification, medication, and bariatric surgery (104). When this framework is applied to pet clinical practice, surgical procedures face significant ethical dilemmas, and there is a gap in the field of medication treatment-there are no officially approved prescription weight-loss drugs for cats to date. Given this, the cornerstone of clinical feline weight management is precise nutritional therapy, which includes prescription food and scheduled feeding, while exercise therapy and behavioral adjustment are core components of the collaborative management. Obesity management is a complex, systematic project, and its success depends on the formulation and continuous implementation of scientific strategies. Multimodal management strategy for obesity in cats is shown in Figure 5.

### 6.1 The principle of weight loss

The weight management strategy for overweight and obese cats has evolved from a broad approach of energy restriction to a more precise and scientific management method. The main principle of this paradigm is that individual weight-loss goals must be established, with the safe and efficient weekly weight-loss percentage of about 0.5 to 2.0 per cent of total body mass (7). Slow weight loss helps in retaining lean mass through adiposity reduction, thus supporting metabolically active tissues, and helps avoid hepatic lipidosis (105, 106). Accurate energy intake calculation underpins the approach; the current recommendation employs a resting energy requirement (RER) formula based on ideal body weight (70 × (ideal body weight kg)^0.75) multiplied by an energy coefficient ranging from 0.8 to 1.0 to determine initial daily intake (37). While caloric restriction is essential, daily energy intake must be adjusted dynamically each week based on weight monitoring data. This adaptive approach helps overcome weight loss plateaus and ensures patient safety throughout the process. Supplemental food should be strictly regulated as part of this type of adaptive approach. Accurate calorie counting must be incorporated into the overall daily energy expenditure calculation. This is particularly important because owners often perceive treats as a bonding item rather than a significant source of calories.

### 6.2 Nutritional approaches

As obligate carnivores, cats have a peculiar metabolism requiring a high level of protein in the diet, which is the primary source for cats to maintain their physiological functions and energy (107). A high-protein, low-carbohydrate diet is particularly beneficial during weight loss, as it promotes satiety, preserves lean body mass, and increases thermogenesis (108, 109). In clinical practice, such diets typically provide 40–50% of metabolizable energy (ME) from protein, 25–35% from fat, and 15–25% from carbohydrates (110). Energy restriction is the cornerstone of weight loss. The initial caloric target is usually calculated as 0.8 × RER based on ideal body weight (e.g., approximately 180–200 kcal/day for a 5-kg cat), with adjustments made every 1–2 weeks based on weekly weight loss of 0.5–2% of body weight (37, 111). Moreover, cats have intrinsically reduced hepatic glucokinase activity, which restricts carbohydrate metabolism (112). Therefore, a low-carbohydrate dietary plan is more consistent with the physiological needs of cats, maintaining post-prandial glucose levels and enhancing insulin sensitivity, which are some of the most crucial mechanisms of preventing and managing diabetes.

In terms of functional ingredients, dietary fiber may decrease food energy density and maintain the well-being of the gut, but the effects of dietary fiber on systemic satiety are controversial (113, 114). Soluble fibers such as psyllium or beet pulp are often included at 2–5% of the diet to promote satiety and glycemic control. The presence of L-carnitine, the vitamin-like compound that helps to transfer the long-chain fatty acids into the mitochondria to be oxidized (115), has proven in human studies to prevent the occurrence of lean tissue loss during weight loss in the form of a supplement (116). In feline weight loss diets, L-carnitine is typically supplemented at 50–100 mg/kg body weight per day to support fatty acid oxidation and preserve lean mass (117). Additionally, omega-3 fatty acids (EPA and DHA) contribute to alleviating obesity-related chronic inflammation (118). Typical inclusion rates in veterinary therapeutic diets range from 0.5–1.5% of dry matter. Therefore, choosing a weight-loss food that has been scientifically formulated, compared to regular “low-fat” cat food, can ensure comprehensive and balanced nutrition as well as functional enhancement while maintaining a low energy intake.

### 6.3 Environmental and behavioral change

Behavioral and management interventions are critical to overcome obstacles to feline obesity management, as they target the root causes of energy excess. Transitioning from ad libitum feeding—a major risk factor for overweight status—to scheduled, portion-controlled feeding is essential. For diabetic cats, consistent meal timing and carbohydrate intake are critical for glycemic control; feeding should ideally coincide with insulin administration to optimize glucose regulation (119). While the optimal meal frequency for weight loss in cats remains an area of investigation, feeding strategies should prioritize alignment with cats’ natural feeding behavior as solitary hunters consuming frequent small meals throughout the day (120). Implementing small, frequent meals—often facilitated by puzzle feeders or automatic feeders—may help stabilize blood glucose and hormonal responses (121) and reduce food-seeking behaviors, although current evidence does not demonstrate that increased meal frequency alone accelerates weight loss (122). The introduction of puzzle feeders and foraging toys is a breakthrough, which makes cats work to get food, which in turn prolongs feeding time and can increase daily energy expenditure by an estimated 10–30%. Additionally, they fulfill the innate predatory instincts, reduce stereotypic behaviors, and alleviate stress (123, 124). Systematic environmental enrichment (e.g., vertical space, resource segregation, and sensory stimuli) supplemented by increased physical activity is also a very valuable supplement to dietary therapy and could prevent weight regain (125). Precision feeding of multi-cat households based on microchip-activated feeders or spatial–temporal segregation can ensure personalized dietary management.

### 6.4 New technologies and pharmacotherapy

The synergistic advancement of technology and pharmacology is reshaping the management paradigm for refractory obesity. Wearable products like digital pet activity trackers are able to measure the level of activity daily to give objective data to gauge compliance with exercise rules (126). Smart scales and feeders are set to compile the food intake and weight variations automatically and create trend charts through applications to enable remote monitoring. Telemedicine portals and pet-health care applications also aid the veterinary follow-ups and data records of the owners, creating a digital system of perpetual assistance (127).

Although there are no feline weight-loss drugs approved, there is the relatively recent advancement of human weight-loss medicines that have significant potential. Glucagon-like peptide-1 (GLP-1) receptor agonists, such as semaglutide and exenatide, mimic the action of endogenous GLP-1. They stimulate insulin secretion, inhibit glucagon release, and delay gastric emptying. These agents are currently used to treat type 2 diabetes and obesity in humans (128). Extensive human clinical trials have shown that these agents reduce the weight in diabetic and non-diabetic patients, with the majority of the patients continuing to lose weight over time (129–131). Although it is not yet formally accepted in cats, small-scale clinical trials and case reports have shown great weight-reduction effects (132). In the future, the creation of veterinary-specific formulations is going to play a very important role, but the existing issues, like the cost, side effects on the gastrointestinal tract, and long-term safety, should be carefully considered.

### 6.5 Client communication and the role of the veterinary healthcare team

The position of the veterinary has changed to that of a conventional diagnosist to that of a lifelong health-management mentor, and the strategies of client-centered communication become a key to success. It has been shown that owners of overweight pets can undergo a lack of awareness about the overweight status of their pet, so far as they even find it more desirable than underweight (133–136). That is why it is necessary to develop standardized protocols that involve the use of body condition and muscle condition scoring at each visit to make monitoring objective (137). Medical workers have to hear out the clients and factor in their needs and limitations into the management strategy. Motivational interviewing, such as the use of open questions and the helpfulness of listening techniques, could help a pet owner realize the problem and advantages of change on their own to develop intrinsic motivation (138, 139). This is an essential strategy because clients have been found to complain when the recommendations received are perceived to be either not suitable or not valuable. Shared establishment of SMART (Specific, Measurable, Achievable, Relevant, Time-bound) objectives like a particular weight-loss objective, via specific, defined activities, gives an opportunity to have a clear roadmap. The process must be accompanied by constant positive reinforcement of every activity of the owner so that confidence and adherence to it can be addressed (140). Another issue that has to be managed concerning the expectations of clients is that they have to be told that the process of losing weight is non-linear and can include plateaus. Introducing a continued support channel with follow-ups (e.g., every 2–4 weeks) and taking the technological platform (e.g., hospital applications) as the means of daily contact would create an environment of sustained support and responsibility, which, in turn, is a key to success in the long run.

### 6.6 Microbiome and metabolomic alterations in obesity

Recent advances in comparative nutrition have highlighted the role of the gut microbiome and metabolomic profiles in feline obesity. Studies have shown that obese cats exhibit distinct microbial compositions compared to lean counterparts, including reduced microbial diversity and alterations in Firmicutes-to-Bacteroidetes ratios (141). These microbial shifts may influence energy harvest, inflammation, and satiety signaling. Weight loss through energy restriction has been shown to induce only minimal changes in fecal microbiota, suggesting that microbial alterations may be more resistant to dietary intervention than previously assumed (141). Metabolomic analyses have further revealed differences in one-carbon metabolism, lipid metabolism, and amino acid profiles between lean and obese cats. For instance, Rankovic et al. (117) reported that serum metabolites related to methionine and cysteine metabolism differ between lean and obese cats, independent of L-carnitine or choline supplementation. Similarly, Grant et al. (142) identified distinct fasting serum metabolomic signatures in obese cats under energy restriction, including alterations in branched-chain amino acids and acylcarnitines, indicating shifts in energy substrate utilization. These findings underscore the potential for using metabolomic and microbiome profiling as tools for phenotyping obesity and monitoring response to weight loss interventions, paving the way for precision nutrition in feline obesity management (143).

## 7 Conclusion and future outlook

Feline obesity is a complex, multifactorial metabolic disorder, not a simple consequence of overfeeding. Its etiology involves an interplay of genetic predisposition, life-stage transitions (notably neutering), and obesogenic environments, culminating in adipose tissue dysfunction that drives chronic inflammation and serious comorbidities such as diabetes and osteoarthritis. This review underscores that effective management requires a paradigm shift: from viewing obesity as a cosmetic issue to treating it as a chronic disease requiring lifelong, medically-supervised intervention. Success hinges on structured programs combining individualized nutritional plans, consistent owner support, and regular monitoring to ensure adherence and prevent relapse. However, despite established management protocols, long-term success rates remain suboptimal, and prevalence continues to rise. To break this stalemate, the field must move beyond incremental improvements and embrace transformative approaches. Future efforts should be strategically directed towards the following paradigm shifts and unresolved challenges:

1. From body condition scoring to “metabolic health phenotyping.”

While BCS is an indispensable clinical tool for diagnosing overweight status, it is a crude surrogate for metabolic health. A critical frontier is the development and validation of dynamic biomarkers (e.g., panels of specific adipokines, lipid species, or gut microbiota metabolites) that can identify the “at-risk” obese phenotype before the onset of insulin resistance or overt inflammation. The research challenge is not to diagnose obesity, but to stratify risk and predict which obese cats will progress to clinical comorbidities, enabling truly preventive, targeted interventions.

2. From generic diets to “predictive nutrigenomics.”

Current “personalized nutrition” often relies on trial and error. The novel opportunity lies in predictive modeling that integrates data on an individual cat’s genotype (e.g., taste receptor variants, metabolic enzyme polymorphisms), baseline microbiome, and metabolic profile to forecast optimal dietary composition. The key research question is: Can we move from retrospective diet adjustment to a prospective, algorithm-driven nutritional prescription that maximizes satiety, metabolic efficiency, and microbiome health from the outset?

3. From adjuncts to “disease-modifying pharmacotherapy.”

The goal of future pharmacotherapy should not merely be to aid weight loss, but to directly target the pathogenic pathways of adipose tissue dysfunction. Novel agents are needed that modulate adipose inflammation, improve adipokine secretion, or enhance lipid turnover without systemic side effects. The unmet need is for therapies that can “re-sensitize” metabolic pathways, making dietary and behavioral interventions more effective, particularly in cats with established metabolic syndrome or severe insulin resistance where diet alone fails.

4. From passive monitoring to “AI-driven adaptive management.”

Current technology (smart feeders, trackers) primarily generates data. The innovation lies in closed-loop, adaptive systems that use artificial intelligence to analyze this data in real-time, predict lapses in adherence, automatically adjust feeding recommendations, and deliver personalized behavioral nudges to owners. The challenge is to transform data into automated, context-aware decision support, reducing the burden on veterinary teams and creating a truly responsive management ecosystem.

5. From lifelong management to “primary prevention via early metabolic programming.”

Prevention is acknowledged but poorly operationalized. A transformative approach requires research into critical windows of susceptibility, such as the post-neutering period or early adulthood. To effectively identify and intervene during these windows, the implementation of regular, lifelong veterinary health check-ups is paramount. Routine physical examinations, including consistent body weight tracking and BCS, are essential for the early detection of subtle weight gain before it progresses to overt obesity. These regular assessments provide the objective data needed to trigger timely preventive interventions, transforming the concept of “early” from a theoretical ideal into a clinical reality. The pivotal question is: Can specific nutritional, environmental, or (in high-risk individuals) pharmacological interventions during these windows “program” a lean, metabolically healthy phenotype for life? This shifts the focus from treating established obesity to preventing its developmental origins.

In conclusion, the next era in feline obesity management must transcend current reactive models. By embracing a focus on metabolic health over weight, prediction over reaction, and pathogenesis over symptomatology, we can evolve towards a future where obesity is not just managed but preempted, dramatically improving feline healthspan and welfare.
